# EQ-5D-3L for Assessing Quality of Life in Older Nursing Home Residents with Cognitive Impairment

**DOI:** 10.3390/life10070100

**Published:** 2020-06-30

**Authors:** Pilar Pérez-Ros, Francisco M Martínez-Arnau

**Affiliations:** 1 Department of Nursing, Universidad Católica de Valencia San Vicente Mártir, 46007 Valencia, Spain; 2 Frailty and Cognitive Impairment Research Group (FROG), University of Valencia, 46010 Valencia, Spain; francisco.m.martinez@uv.es; 3 Department of Physiotherapy, Universitat de València, 46010 Valencia, Spain

**Keywords:** cognitive function, nursing home residents, quality of life, EQ5D, measurement properties

## Abstract

Background: Quality of life (QoL) is recognized as an important patient-reported outcome measure. Assessing QoL in older people with cognitive impairment is a challenge due to discrepancies in the collection of data via proxies versus self-report. This study aimed to assess the psychometric properties of the self-reported EQ-5D (including the EQ index and EQ visual analog scale (VAS)) in nursing homes residents with cognitive impairment and to analyze its validity based on scales included in the comprehensive geriatric assessment. Methods: Cross-sectional, multicenter study analyzing the feasibility, acceptability, reliability, and validity of the EQ-5D based on 251 self-administered questionnaires in a sample of nursing home residents with cognitive impairment. Reference scales were those from the comprehensive geriatric assessment, equivalent to the five dimensions of the EuroQol. Results: The EQ index was 0.31 (0.37) and the EQ VAS was 35.96 (29.86), showing adequate acceptability and feasibility. Cronbach’s alpha was 0.723. The EQ index and EQ VAS, as outcome variables for multiple linear regression models including CGA titration scales, showed better validity for the EQ index than the EQ VAS. Conclusions: As a self-administered generic scale, the EQ-5D-3L could be a good tool for QoL assessment in nursing home residents with cognitive impairment.

## 1. Introduction

The world population has rapidly aged in the last 50 years due to falling birth rates and rising life expectancy. In older populations, physical and mental health-related problems are directly related to perceived quality of life (QoL). Older people wish to live longer but also with good QoL [1]. The increase in the older population correlates to a greater need for social and healthcare resources, making it necessary to identify the most effective interventions for enhancing older people’s quality of life while providing the best value for money. Analyzing quality of life is useful for comparing the cost-effectiveness of different interventions [2].

The World Health Organization (WHO) describes QoL as “a broad ranging concept affected in a complex way by the person’s physical health, psychological state, level of independence, social relationships, personal beliefs, and their relationship to salient features of their environment” [3]. Other factors that influence the perception of QoL encompass dimensions related to gender, comorbidity, loss of mobility, depression, and dependency [1,2]. 

There are several generic scales for assessing QoL in older people living both in the community and in institutions, as well as specific scales according to different comorbidities [4,5]. This great variety of instruments is accompanied by an overall lack of standardization, but there is consensus that the best QoL instrument should be generic, feasible, brief, multidimensional, and subjective [1,2].

Cognitive impairment and dementia comprise one of the most important geriatric syndromes due to its chronic and insidious nature, which directly affects older people’s quality of life [6]. There is some controversy about the capacity of people with cognitive impairment to undergo an evaluation of their quality of life. Some authors indicate that people’s intellectual deterioration incapacitates them for this type of subjective assessment, and they adopt an indirect focus using proxies; that is, the main caregivers (either family members or professionals in the institution where they live) undertake the assessment for them. In contrast, other authors note that people with cognitive impairment are aware of the progressive loss of their capacities, so it is appropriate to evaluate their QoL directly, as this impairment is reflected in the outcome [7]. 

Many studies have investigated the factors that influence QoL in people with dementia. The perceived loss of cognitive capacity decreases QoL, as does the loss of independence [8]. People living in institutions generally have a high prevalence of cognitive impairment, morbidity, and loss of function and mobility. In addition, they may be affected by the lack of socialization in institutional settings, environmental factors like distance from home, and difficulties adapting to the nursing home. So nursing home residents tend to be more affected by impaired quality of life than community-dwelling older people [9,10,11].

In a systematic review of various QoL scales in nursing homes, authors conclude that the QUALIDEM scale [12,13] is the most advisable for residents with dementia and the Psychosocial Quality-of-life Domains Questionnaire [14] for those without [5]. At the same time, the National Institute for Health and Care Excellence (NICE) recommends using the EuroQoL-5 Dimensions (EQ-5D) for economic evaluations of healthcare interventions [15]. In addition to being the most widely used scale worldwide, with translations to 180 languages, it is brief and easy to use. The two-part scale includes a descriptive section for calculating the EQ-5D index, which includes dimensions evaluating mobility, self-care, usual activities, pain, and anxiety, as well as a visual analog scale (VAS) [16]. The EQ-5D is a self-administered questionnaire that does not consider cognition in people with impaired faculties, so an evaluation of the cognitive dimension was subsequently added. This amendment improved the content validity, while the reliability remained unaltered [17]. 

Diaz-Redondo et al. analyzed the psychometric properties of the proxy-rated EQ-5D, showing that it is a valid alternative for assessing quality of life in institutionalized older people with dementia [18]. Ankri et al. [7] also point to the possibility of using the EQ-5D for people with dementia, although different authors indicate the need for more studies that analyze both its properties for assessing QoL according to the severity of the dementia and its validity in the absence of a gold standard [4,7].

The most widely used geriatric evaluation tool is the comprehensive geriatric assessment (CGA), which consists of a complete geriatric history and a physical and cognitive examination, including geriatric assessment scales that facilitate an accurate clinical, functional, psychological, and social examination [19]. The scales comprising the CGA assess all the dimensions included in the EQ-5D as well as others that are not. The functional domain evaluates the capacity to perform basic and instrumental activities of daily living (ADLs and IADLs) and the ability to walk around. In addition, the psychological domain assesses cognition and the emotional state, and the social domain includes items on loneliness and social resources accessible to the older person [19,20,21]. 

Comparing outcomes obtained from different scales is difficult, both in community and institutional settings. Given that the EQ-5D is brief, user-friendly, and commonly used in communities, the aim of our study was to assess the psychometric properties of the EQ-5D in nursing homes residents with cognitive impairment and to analyze its validity based on scales included in the CGA.

## 2. Materials and Methods 

### 2.1. Study Design and Participants

A cross-sectional study was carried out from January 2020 to March 2020 in seven nursing homes for older adults in the province of Valencia. The inclusion criteria were: participants aged 70 years or older; cognitive impairment diagnosed by geriatrician after cognitive evaluation with the Mini Mental State Examination (MMSE)**,** with Cronbach’s alpha 0.90 and a score of 10 to 24, indicating cognitive impairment [22,23]; living in a nursing home in the province of Valencia (Spain). Exclusion criteria were: refusal to participate in the study, the existence of associated disease conditions resulting in a life expectancy of under six months, blindness and deafness, serious psychiatric problems (severe depression subjected to treatment or acute psychosis), or severe cognitive impairment (diagnosed previously by a physician). 

### 2.2. Sample Size Description

The sample size was calculated based on the population census of 2019, showing that 15,145 older people were living in nursing homes province-wide. A sample of 195 participants was required to estimate a 27% incidence of older adults with mild-to-severe cognitive impairment, with an alpha error of 5%, precision of 3%, and a statistical power of 95%. 

In order to use an adequate sampling frame, we decided to recruit participants over a period of three months. The research team visited the nursing homes and hung posters to inform the residents and their relatives about the study objectives. Volunteers signed up on a list in each center, and their data were recorded using alphanumeric codes identifying the center and the individual. All people who showed their willingness to participate and who met the inclusion criteria were included. 

#### 2.2.1. Data Collection and Quality of Life Assessment

Personalized interviews were undertaken with each participant to perform CGAs and collect data on age and sex as well as functional, cognitive, and emotional variables. The four nurses in charge of this task had at least five years’ experience in primary health care centers and nursing homes; they were not involved in the study and were unaware of its objectives.

Health-related quality of life was measured using the EQ-5D index and the EQ-5D VAS, according to the parameters of the Spanish population. The EQ-5D-3L (Levels) version has been translated into over 140 languages, and it provides information on three aspects of QoL. First, its descriptive system assesses the level of impairment in each of the five dimensions included in the scale: mobility, self-care, usual activities, pain/discomfort, and anxiety/depression. Each dimension has three levels of impairment: no problems (level 1), some problems (level 2), and extreme problems (level 3). Second, the descriptive response from the EQ-5D can be converted into an index score. The score ranges from less than 0 to 1, (where 0 is a health state equivalent to death and negative values are worse than death) and 1 is the most positive score (the maximum level of perceived QoL according to the five dimensions included on the scale). Finally, the EQ VAS score is obtained by asking the patients to rate their health on a 20-cm vertical scale. The scale ranges from 0 to 100, where 0 is the “worst imaginable health” and 100 is the “best imaginable health” [16].

The functional and emotional assessment tools were the Tinetti Balance and Gait Scale, the Barthel Index Basic Activity of Daily Living, Lawton Instrumental Activity of Daily Living Scale, Yesavage Geriatric Depression Scale (GDS), and the VAS pain. These tools can be part of the CGA or used in isolation. In the present study, they were used to obtain the information and complete an individualized CGA for each participant.

The Tinetti index consists of two parts (28 points total): the first seeks to assess balance, and it has nine items, totaling 16 points; the second corresponds to gait assessment, and it has eight items, totaling 12 points. A total score of less than 19 points indicates a fivefold increased risk of falls, so the lower the total score, the higher the risk of falls [24].

The Barthel Index (BI) consists of 10 items that measure a person’s daily functioning, particularly the ADLs. The items include feeding, transfers (from bed to chair and back, and to and from the toilet), grooming and toileting, walking on a level surface, going up and down the stairs, dressing, and bowel and bladder continence. The total score ranges from 0 (totally dependent) to 100 (totally independent) scores are awarded in multiples of 5 [25].

The Lawton IADL scale assesses people’s ability to perform eight activities (using the telephone, shopping for groceries, food preparation, housekeeping, laundering, self-medicating, transportation, and managing finances). The total score ranges from 0 (totally dependent) to 8 (totally independent) [26].

The Short Form GDS consists of 15 questions: 10 indicate the presence of depression when answered positively, while the rest (questions 1, 5, 7, 11, 13) indicate depression when answered negatively. Scores of 0 to 4 are considered normal, depending on age, education, and complaints; 5 to 8 indicate mild depression; 9 to 11, moderate depression; and 12 to 15, severe depression [27].

The VAS pain scale [11] is a continuous scale comprised of a horizontal or vertical line, usually 10 cm in length. For pain intensity, the scale goes from 0 (indicating no pain) to 10 (worst imaginable pain) [28].

#### 2.2.2. Measurement Properties

We analyzed the main measurement properties of the QoL instruments, including feasibility, acceptability, reliability, and construct validity, according to the criteria set out in Table 1. As there is no gold-standard measure for QoL, criterion validity was not appraised [5].

Feasibility indicates the capacity to use the instrument under normal conditions, so we analyzed the percentage of missing data (should be <10%) and computable data (should be >95%) [29]. Acceptability indicates an adequate distribution of scores among the sample [30]; to assess this property, we calculated the difference between the mean, the median, and the standard deviation (SD) (allowing a divergence of 15%); the existence of the floor and ceiling effect in both the dimensions like the EQ index and the VAS (extreme scores should comprise <15% of the total) [31]; and finally, the asymmetry (acceptable limits between −1 and 1) [32]. To analyze reliability, we assessed internal consistency and the stability of the measure. Internal consistency was calculated using Cronbach’s alpha (acceptable values ≥ 0.7) [31]; stability was measured using the intraclass correlation coefficient (ICC) between the EQ index and the EQ VAS (one-way, random-effects model; acceptable values were ≥0.7). ICC values of less than 0.5 were indicative of poor reliability; 0.50 to 0.74, moderate reliability; 0.75 to 0.90, good reliability, and more than 0.90, excellent reliability [33].

Convergent validity determines the relationship of the scale with other measures assessing the same construct. The technique used was the correlation coefficient (Pearson’s or Spearman’s), which was assessed according to Feeny et al.’s criteria: high correlation, r ≥ 0.50; moderate, r 0.35 to 0.49; and weak, r ≤ 34 [34]. In this way, we analyzed the correlation between the EQ index and the EQ VAS. The results of each EQ-5D domain were rated from 1 (no problems) to 3 (extreme problems) and compared to the scales assessing the same domains. The Tinetti scale functional score refers to domain 1 (mobility) in the EQ-5D; the Barthel scale, to domain 2 (self-care); the Lawton scale, to domain 3 (usual activities); the GDS, to domain 4 (anxiety-depression); and the VAS pain scale, to domain 5 (pain). 

In order to relate the quantitative scales with the EQ-5D domains, we classified the validated scores of each scale into three categories, corresponding to the EQ-5D levels 1 to 3. Thus, for mobility, the Tinetti scores were categorized as follows: level 1 (no problems), 20 to 28 points; level 2 (some problems), 10 to 19 points; and level 3 (extreme problems), 0 to 9 points. For self-care, the distribution on the Barthel Index was: level 1, 65 to 100 points; level 2, 35 to 60 points; and level 3, 0 to 30 points. For usual activities, Lawton scores were classified as: level 1, 6 to 8 points; level 2, 3 to 5 points; and level 3, 0 to 2 points. For pain/discomfort, VAS scores were divided into: level 1, 0 to 3 points; level 2, 4 to 7 points; and level 3, 8 to 10 points. For anxiety/depression, the GDS scores were translated as: level 1, 0 to 4 points; level 2, 5 to 10 points; and level 3, 11 to 15 points.

Divergent validity refers to the association between the scale and other measures that assess different constructs. To analyze this property between the EQ-5D (Index and VAS) and the CGA, we used the correlation coefficient, which hypothetically should be low (r ≤ 0.30) [35].

Finally, to assess internal validity, two linear regressions were performed with the EQ index and the EQ VAS, including the scales categorized into the three levels equivalent to the dimensions of the EQ-5D-3L, in order to understand the relationship between the variables included and the value of R^2^.

#### 2.2.3. Statistical Analysis

Variables were reported as proportions and/or means and SDs. The Kolmogorov–Smirnov test was used to assess normality, and the Levene test was applied to explore homogeneity of variances for continuous variables (age, MMSE, EQ-5D VAS and EQ-5D index, Barthel Index, Lawton Index, Tinetti Index, GDS, and VAS pain). There were no significant outliers. The data met the main assumptions of normality, so the t-test for independent samples was used to compare means. The chi-squared test was used to compare categorical variables (gender). 

## 3. Results

Of the 310 eligible participants, 45 did not wish to take part, and 14 were not capable of responding to the items on the questionnaire. The final sample was 251 nursing home residents, with a predominance of women (76.9%; *n* = 193). The group presented substantial levels of dependence in the ADLs according to the Barthel and Lawton indexes, gait problems (Tinetti), and depressive symptoms (GDS). In contrast, the mean pain assessment showed a value of less than 3 (Table 2).

**Figure 1:**
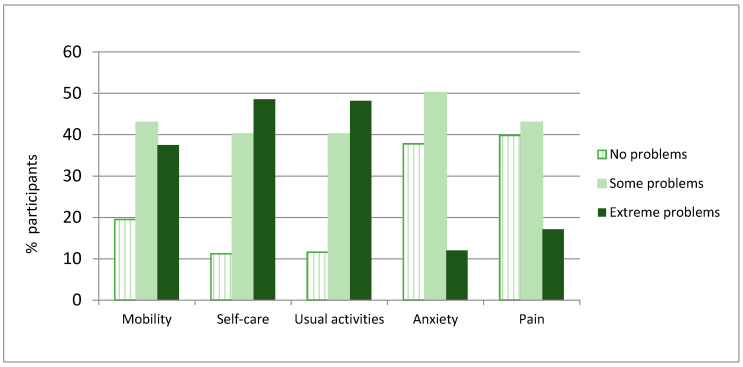
Distribution of responses on the EuroQol dimensions.

The participants presented low QoL scores on both the EQ index and the EQ VAS, with mean index and VAS scores hovering around 30% of the maximum possible QoL (Table 3). In the analysis of each dimension, there was a high prevalence of extreme problems in self-care (48.6%, *n* = 122) and usual activities (48.2%, *n* = 121). A lower proportion had major problems in mobility (37.5%, *n* = 94), pain (17.1%, *n* = 43), and anxiety (30%, *n* = 12) (Figure 1).

### 3.1. Psychometric Properties: Feasibility and Acceptability

Feasibility was adequate: there were no missing data, and just 5.3% (*n* = 14) of the 265 older nursing home residents who were willing to participate were unable to respond to the questionnaire items.

Likewise, the values for the acceptability of the EQ index were within the recommended bounds. However, a ceiling effect and kurtosis of more than −1 were seen on the EQ VAS (Table 3). 

### 3.2. Psychometric Properties: Reliability and Validity

In the analysis of internal consistency as a measure of reliability, we observed an acceptable value for Cronbach’s alpha, of 0.723. To assess the stability of the measure, we calculated the ICC for the EQ index and the EQ VAS, obtaining a value of 0.01 (95% confidence interval [CI] −0.26 to 0.23; *p* = 0.452), indicating a poor correlation. 

To determine the convergent validity, we analyzed the correlation between the five dimensions of the EQ and the corresponding scales included in the CGA (Tinetti, Barthel, Lawton, GDS, and VAS pain), categorized into three levels as indicated in the Methods. The correlation was high for dimension 2, moderate for dimension 3, and low for dimensions 1 and 5. No correlation was observed for dimension 4 (Table 4).

**Figure 2:**
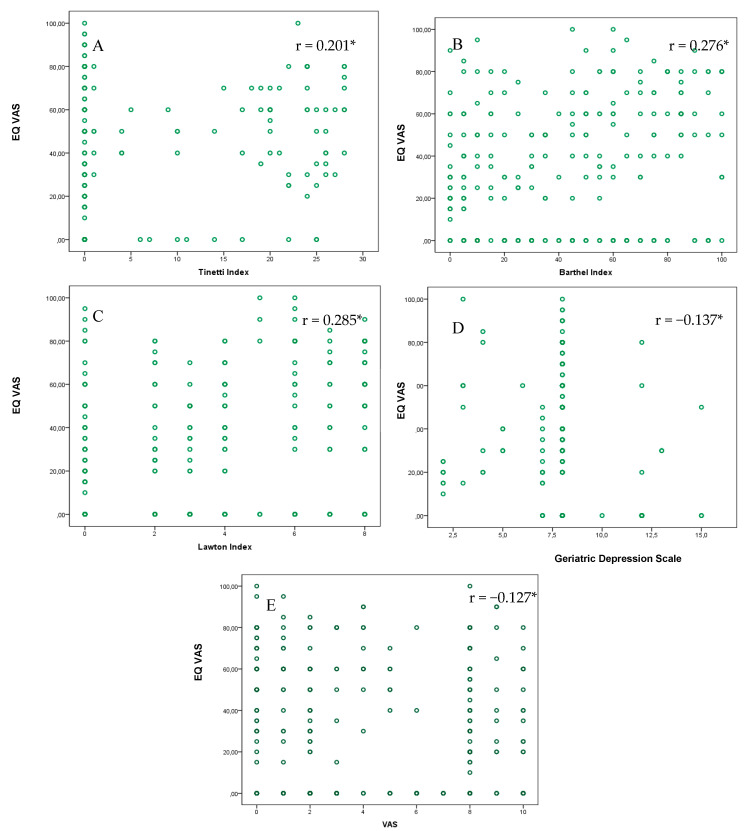
Scatter plot between the EQ VAS and the scales included in the Comprehensive Geriatric Assessment. (**A**) Tinetti Index (0–28: lower scores = more dependence in mobility). (**B**) Barthel Index (0–100: lower scores = more dependence in ADLs). (**C**) Lawton Index (0–8: lower scores = more dependence in IADLs). (**D**) The Geriatric Depression Scale GDS (0–10: higher scores = more depression). (**E**) The visual analog scale VAS (1–10: higher scores = more pain). * *p* < 0.001; ** *p* < 0.05

To assess the divergent validity, we analyzed the correlations between continuous values of the EQ index and the EQ VAS, on the one hand, and the corresponding scales included in the CGA, on the other. As shown in Figure 2, there were low, statistically significant correlations between the EQ VAS and all of the scales of the CGA. 

**Figure 3:**
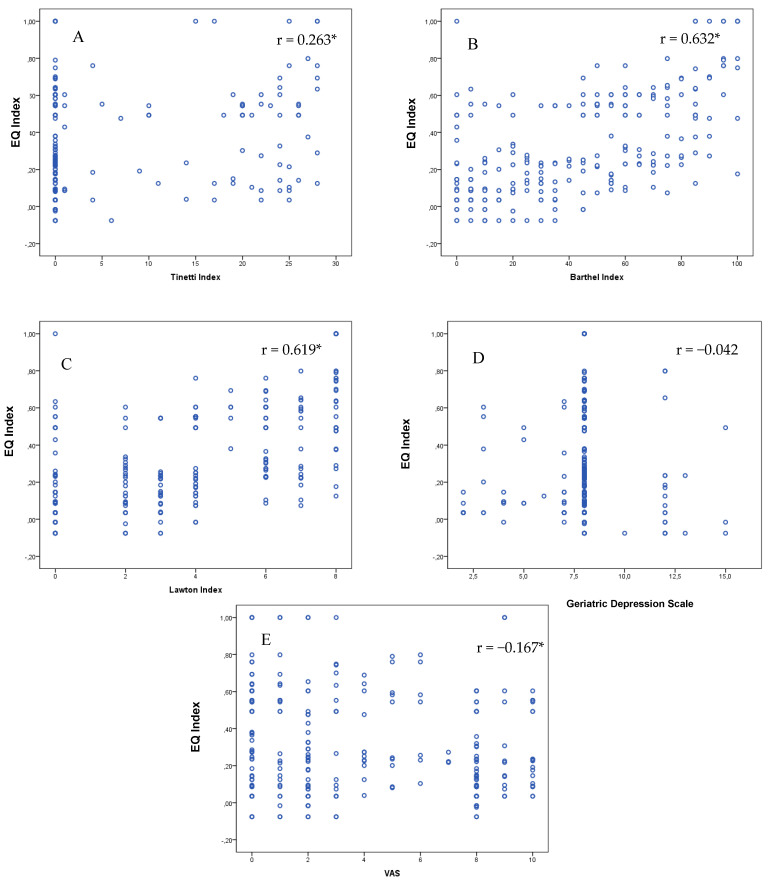
Scatter plots between the EQ index and the scales included in the Comprehensive Geriatric Assessment. (**A**) Tinetti Index (0–28: lower scores = more dependence in mobility). (**B**) Barthel Index (0–100: lower scores = more dependence in ADLs). (**C**) Lawton Index (0–8: lower scores = more dependence in IADLs). (**D**) The Geriatric Depression Scale GDS (0–10: higher scores = more depression). (**E**) The visual analog scale VAS (1–10: higher scores = more pain). * *p* < 0.001; ** *p* < 0.05

As shown in Figure 3, there were high correlations between the EQ index and the Barthel and Lawton scales. Significant correlations—albeit with lower r values—were also observed with the Tinetti scale and the VAS pain. In contrast, there was no correlation for depression symptoms assessed using the GDS versus the EQ index (Figure 3). 

Finally, we performed two linear regressions, using the EQ index and the EQ VAS as outcome variables and the scores of the scales included in the CGA, categorized in three levels and adjusted for age and sex, as explanatory variables. For both the EQ index and the EQ VAS, the model showed a statistically significant relationship (EQ index: F = 18.017; *p* < 0.001; R = 0.625; R^2^ = 0.39; EQ VAS: F = 2.967; *p* = 0.006; R = 0.309; R^2^ = 0.095). Impaired function for performing the ADLs, as assessed by the Barthel Index, was the variable that was most strongly (and negatively) correlated with both measures (Table 5). 

## 4. Discussion

The increase in life expectancy among the older population has been accompanied by a rise in the prevalence of geriatric syndromes like fragility and cognitive impairment [2]. Assessing quality of life in older people with cognitive impairment is still a challenge because the use of evaluation instruments is not standardized, and moreover, the methods for data collection vary according to whether the older person or the caregiver answers the questionnaire. This study assessed the measurement properties of the EQ-5D as a self-report tool in older nursing home residents with cognitive impairment. Our results show that the descriptive system and EQ index present good validity and reliability, while validity decreases with the EQ VAS.

The presence of comorbidity and different settings of residence (home versus institutions) complicates the choice of the most appropriate instrument for assessing QoL in older people, although generic assessment tools are recommended [2,36]. Older nursing home residents with cognitive impairment have worse QoL than community-dwelling older people, in large part due to functional loss in mobility and in the ADLs [37,38,39]. This aspect was confirmed in our results, with participants showing dependence in performing IADLs and in mobility but no great health impact due to pain or anxiety/depression. 

Understanding the psychometric properties of the EQ-5D in older people with cognitive impairment can shed light on the appropriateness of a generic scale that is already widely used in community settings [40]. The feasibility of the tool in practice and its acceptability among users was adequate [29,30,31,32], perhaps because the EQ-5D is easy to use, generic, and brief. Although there are other dementia-specific scales that assess numerous items, some do not present good measurement properties [5,36]. In part, these limitations could be because assessing QoL in older people with cognitive impairment poses challenges [41], including the appearance of fatigue after a long evaluation or frustration if the instrument probes areas where the older person is aware of their limitations. Such experiences could lead patients to abandon the assessment. 

Reliability and validity were also good [33,34,42]. The CGA is a powerful tool for anamnesis in older people [21], bringing together the clinical, functional, emotional, and social dimensions that converge in this population. The same dimensions are addressed in the EQ-5D [16]. On comparing the two instruments, a good R^2^ value was observed, which provides information on the validity of the latter scale. The absence of a gold standard [5] complicates the identification of comparison measures, but in our case we obtained acceptable results in the regression performed for the EQ index, although less so for the EQ VAS. Diaz Redondo et al. highlighted this last aspect [18], although they used proxies. Thus, using the dimensions and calculating the EQ index with the EQ-5D could be a valid method for assessing QoL in nursing home residents with cognitive impairment. Obtaining the EQ VAS score alone would not afford the same validity, although the information it contributes could complement that provided by the five dimensions.

The results of the present study indicate that self-reported EQ-5D in nursing home residents with cognitive impairment could be a good instrument for assessing QoL, as it is generic, brief, and easy to use. A review by Yang et al. recommends using generic scales like the EQ-5D by proxy in older people with dementia [36], and Diaz-Redondo et al. [18] obtained good ICCs with the proxy-reported EQ-5D in older nursing home residents with dementia, despite the low internal consistency, the lack of unidimensionality, and the presence of ceiling and floor effects for some dimensions. Notwithstanding the evidence provided by several reviews, there is a dearth of studies in this population analyzing self-perceived QoL, which is a much more faithful measure than that obtained via proxies [2]. Aspden et al. [5] also analyzed the psychometric properties of instruments used in nursing home residents, finding numerous gaps in the consistency and reliability of the tools. The authors conclude that the choice of assessment instruments depends on the presence or absence of dementia, calling for further research on the topic. Specific scales exist to assess self- and proxy-perceived QoL in people with dementia, for example the Quality of Life in Alzheimer’s Disease (QOL-AD), which has shown good measurement properties [43,44]. However, using a more generic scale would enable comparisons between results from different settings (communities versus institutions) and from different populations (with versus without cognitive impairment). 

The present study has some limitations. First of all, interobserver validity could not be assessed, as this was a self-administered questionnaire. Likewise, our research team opted not to ask participants to fill in the questionnaire a second time in order not to overburden their caregivers, even though the EQ-5D assesses the perceived health state in the moment that the scale is used, and this could vary if completed on different days. Moreover, we did not consider participants’ comorbidities or the social dimension within the nursing home environment. Finally, results may differ if groups are analyzed according to ranges of MMSE scores, so more analysis is needed.

## 5. Conclusions

The psychometric properties of the EQ-5D as a self-administered scale in nursing home residents with cognitive impairment are adequate. Compared to the scales included in the CGA, internal validity is good for the EQ index but less so for the EQ VAS. The use of this generic instrument for assessing QoL in this population of older people could be an adequate option. 

## Figures and Tables

**Table 1:** Measurement properties.

Property	Criteria
Feasibility	Percentage of missing data (should be <10%) Percentage of computable data (should be >95%)
Acceptability	Mean, median, and standard deviation similar across items (15% maximum divergence) Asymmetry and kurtosis should oscillate between −1 and 1 Floor and ceiling effect (percentage of scores in the lower and upper extremes; should be <15%)
Reliability	Internal consistency: Cronbach´s alpha (≥0.7) Stability of the measure: ICC (EQ index and EQ VAS)
Validity	Convergence: correlation between EQ index and VAS, and correlation between dimensions of EQ and assessment scales Divergence: correlation between EQ index and EQ VAS and assessment scales Internal: R^2^ of two linear regressions between EQ index and assessment scales and EQ VAS and assessment scales

ICC: intraclass correlation coefficient; EQ index: EuroQol 5 Dimensions 3 Levels Index; EQ VAS: EuroQol Visual Analog Scale.

**Table 2:** Baseline participant characteristics.

Variables	Mean (SD)	Min	Max
Age, years	84.6 (9.22)	70	104
MMSE, points (0–30)	15.60 (5.23)	10	24
Barthel Index, points (0–100)	41.10 (31.38)	0	100
Lawton Index, points (0–8)	3.57 (2.90)	0	8
Tinetti Index, points (0–28)	6.58 (10.19)	0	28
GDS, points (0–15)	7.88 (2.02)	2	15
VAS pain, points (0–10)	1.8 (0.91)	0	10
EQ VAS (0–100)	35.96 (29.86)	0	100
EQ index (less than 0–1)	0.31 (0.37)	0	1

EQ: EuroQoL; GDS: Geriatric Depression Scale; MMSE: Mini Mental State Examination; SD: standard deviation; VAS: visual analog scale.

**Table 3:** Properties regarding the feasibility and acceptability of the EQ index and EQ VAS.

	EQ Index	EQ VAS
Mean	0.31	35.96
Standard deviation	0.37	29.89
Median	0.23	35
Asymmetry	0.69	0.187
Kurtosis	−0.32	−1.239 *
Ceiling	4.4	29.9 ^†^
Floor	4.4	0.8

* Kurtosis more than −1; ^†^ Ceiling effect > 10%.

**Table 4:** Convergent validity. Correlation between EuroQol 5 Dimensions and comprehensive geriatric assessment scales.

EuroQol 5 Dimensions	Comprehensive Geriatric Assessment Scales	*r*
1. Mobility	Tinetti Index	−0.215 *
2. Self-care	Barthel Index	−0.606 *
3. Usual activities	Lawton Index	−0.475 *
4. Pain	VAS pain	0.094
5. Anxiety	Geriatric Depression Scale	0.179 **

VAS: visual analog scale; * *p* < 0.001: ** *p* < 0.01.

**Table 5:** Multiple linear regression analysis with EQ-5D Index and VAS as dependent variables.

	EQ Index β (95% CI)	*p*	EQ VAS β (95% CI)	*p*
Constant	0.54 (0.41, −0.68)	<0.001	53.02 (35.25, 70.78)	<0.001
Barthel	−0.17 (−0.26, −0.08)	<0.001	−13.31 (−25.32, −1.31)	0.030
Lawton	−0.03 (−0.11, 0.06)	0.558	5.54 (−5.76, 16.84)	0.335
Tinetti	−0.01 (−0.04, 0.04)	0.950	−4.42 (−9.63, 0.78)	0.096
VAS	−0.03 (−0.06, 0.01)	0.084	0.18 (−4.31, 4.67)	0.938
GDS	0.04 (−0.05, 0.13)	0.369	−2.55 (−14.36, 9.26)	0.671

CI: confidence interval; GDS: Geriatric Depression Scale; VAS: visual analog scale.
